# 血清多肽组学作为Ⅰ期-Ⅱb期非小细胞肺癌标志物的初步研究

**DOI:** 10.3779/j.issn.1009-3419.2019.01.05

**Published:** 2019-01-20

**Authors:** 跃龙 侯, 洪琦 郭, 永宽 郭, 玉坤 张, 洪利 韩

**Affiliations:** 300170 天津，天津市第三中心医院胸外科 Department of Thoracic Surgery, Third Central Hospital of Tianjin; Tianjin Institute of Hepatobiliary Disease; Tianjin Key Laboratory of Artificial Cell; Artificial Cell Engineering Technology Research Center of Public Health Ministry, Tianjin 300170, China

**Keywords:** 血清多肽组, 肺肿瘤, 肿瘤标志物, 早期诊断, Serum peptidomics, Lung neoplasms, Tumor biomarker, Early diagnosis

## Abstract

**背景与目的:**

非小细胞肺癌是发病率最高的一种肺恶性肿瘤，早发现、早诊断、早治疗是其治疗原则，因其发病隐匿，缺乏早期筛查的特异性标志物，多数患者就诊时已处于晚期阶段，导致其5年生存率较低，预后不理想。探索一种敏感的生物学标志物，是目前肺癌诊疗的重点。本文基于差异多肽组学技术，旨在筛选Ⅰ期-Ⅱb期非小细胞肺癌的血清生物标志物。

**方法:**

利用纳升超高效液相色谱联合四级杆轨道阱质谱技术，比较和筛选正常健康人群、肺部良性病变患者、非小细胞肺癌患者血清中的差异多肽片段，通过对目标肽段的鉴别和定量分析，探寻非小细胞肺癌早期诊断的肿瘤生物标志物。

**结果:**

通过与人类蛋白质组数据库进行比对，共鉴定到545个多肽，分别来自118个蛋白。将各组样本中肽段的谱图数进行比较，共筛选出201个差异多肽，经定量分析发现峰面积的变异系数（coefficient of variation, CV）≤30%的目标肽段共计7个，其中在各组间呈趋势变化的有2条肽段，分别是肺癌组中表达下调的肽段QGAKIPKPEASFSPR和肺癌组中表达上调的肽段CDDYRLC，它们分别来自于中间α球蛋白抑制因子H4蛋白（ITIH4）和基质γ-羧基谷氨酸蛋白（MGP）。

**结论:**

将血清多肽组学用于筛选肿瘤标志物的研究，可为非小细胞肺癌的早期诊断提供新线索，其中ITIH4与MGP蛋白水解的特异性肽段是潜在早期非小细胞肺癌患者的血清标志物。

## Abstract (en)

**Background and objective:**

Non-small cell lung cancer (NSCLC) have the highest incidence of lung cancer which treatment principles are diagnosis and treatment as early as possible. Because of its insidious onset and lack of specific markers for early screening, most patients are at an advanced stage when diagnosed which results in a low 5-year survival rate and poor prognosis. Therefore Exploring a sensitive biomarker is the focus of current diagnosis and treatment of lung cancer. The aim of this study is to investigate the biological markers in serum of patients with Ⅰ-Ⅱb stage NSCLC by differential peptidomics analysis.

**Methods:**

The serum peptidome was compared and analyzed among the groups of normal health controls, benign lung diseases and early stage NSCLC patients using a nano ultra-performance liquid chromatography combined with a quadrupole-orbitrap mass spectrometer. The differentially expressed polypeptides were identified and analyzed quantitatively to screen the tumor biomarkers for the early diagnosis of NSCLC patients.

**Results:**

According to the Swiss-Prot database, a total of 545 polypeptides originated from 118 proteins were identified. The spectral numbers of serum polypeptides in each group were compared and a total of 201 polypeptides differentially expressed were found. Following a quantitative analysis of the above peptides, we found that there were 7 peptides with the coefficient of variation (CV) less than 30% and among them the peptide of QGAKIPKPEASFSPR from ITIH4 was down-regulated and the peptide of CDDYRLC from MGP was up-regulated in NSCLC group.

**Conclusion:**

The tumor biomarkers obtained by serum peptidome technology can provide a new clue for early diagnosis of NSCLC and the specific peptides hydrolyzed from ITIH4 and MGP may be the serum biological markers for early NSCLC patients.

非小细胞肺癌（non-small cell lung cancer, NSCLC）是最为常见的一种肺癌类型，其发病率约占肺癌患者的85%^[1]^。由于NSCLC发病隐匿，缺乏早期筛查的特异性标志物，多数患者就诊时已处于晚期阶段，常伴随淋巴结及远处转移，导致其5年生存率较低，预后也不理想。研究认为，通过对NSCLC高危人群进行有效筛查，确立早期NSCLC发病人群并采取有效的治疗措施，可以显著提高NSCLC病患的生存率，改善其预后^[2]^。多年来，大量的科学研究关注于多种肿瘤生物标志物的探索和开发^[3-5]^，但目前临床对于早期NSCLC患者的筛查，尚缺乏特异性标志物的辅助诊断。

多肽组学是一种分析多肽链结构、功能及其变化规律的组学技术，是蛋白1组学的深入补充。近年来，越来越多的科研工作者尝试利用多肽组学技术，对各类疾病进行早期筛查和预后评估，并发现了一系列生物标志物^[6-8]^。因此，本文旨在通过血清多肽组学分析技术，比较正常健康人群、肺部良性病变患者、早期NSCLC患者血清中的差异多肽片段，筛选和鉴定早期NSCLC患者的肿瘤生物标志物，从而以新的角度为NSCLC的早期诊断提供线索和依据。

## 1 材料与方法

### 1.1 一般资料

收集2016年9月-2017年9月天津市第三中心医院住院收治的27例初诊NSCLC早期患者的血清样本，患者中位年龄61.5岁（50岁-74岁），其中男性17例、女性10例，肺癌术后TNM分期：Ia期-Ⅱb期，分型包括12例鳞癌、12例腺癌、1例腺鳞癌、2例大细胞癌。同时收集26例肺部良性病变患者的血清样本，年龄54.7岁（36岁-69岁），其中男性16例、女性10例，良性病类型包括肺囊肿、结核球、机化性肺炎、硬化性血管瘤、炎性假瘤、错构瘤、肉芽肿炎及非典型腺瘤样增生。另外采集14例健康体检人群的血清样品，年龄52（21岁-59岁），其中男性8例、女性6例（表 1）。所有NSCLC组和良性组患者在血清样本采集前均未进行手术、放化疗及介入等治疗，且术后均得到明确的病理学证实。所有纳入实验的研究对象均已签署知情同意书。

**1:** 各组受试者的临床病例特征 Clinical and pathologic characteristics in each group

Clinical features	Cancer			Benign			Normal		*P*
	*n*	%		*n*	%		*n*	%	
Gender									0.795
Female	10	37.1		10	38.5		6	42.9	
Male	17	62.9		16	61.5		8	57.1	
Age (yr)									0.185
≥60	17	62.9		12	46.2		5	35.7	
< 60	10	37.1		14	53.8		9	64.3	
Smoking history									0.265
Yes	11	40.7		11	42.3		8	57.1	
No	16	59.3		15	57.7		6	42.9	
Classification									
SCC	12	44.4							
AC	12	44.4							
ASC	1	3.8							
LCC	2	7.4							
pTNM stage									
ⅠA	14	51.9							
ⅡA	5	18.5							
ⅡB	8	29.6							
SCC: squamous cell carcinoma; AC: adenocarcinoma; ASC: adenosquamous carcinoma; LCC: large cell carcinoma; TNM: tumor node metastasis.									

### 1.2 样本分组与前处理

将血清样本分为三组：分别为正常对照组（Normal组）、良性病变组（Benign组）、早期NSCLC组（Cancer组），根据每组的样本数，进行多样本混合，使每组有3个混合生物样本（表 2），取混合好的样本50 μL加入等量的标准肽，而后加入200 μL的20%乙腈（含50 mmol/L NH_4_HCO_3_），4 ℃静置5 min，将样本过10 kD滤膜后，低温超速离心30 min（4 ℃、10, 000 g），加入终浓度5 mmol/L二硫苏糖醇（DTT），30 ℃震荡30 min，加入10 mmol/L碘乙酰胺（IAM），避光保存45 min，使用C18 tip柱对样本进行除盐，真空离心抽干，使用液相流动相A复溶，离心后转移至样品瓶中，待测。

**2:** 实验分组与样本混合 Experiment grouping design and mixed samples

Group	Replication	Sample counts
Normal	Normal 1	5
	Normal 2	5
	Normal 3	4
Benign	Benign 1	9
	Benign 2	9
	Benign 3	8
Cancer	Cancer 1	9
	Cancer 2	9
	Cancer 3	9

### 1.3 液相色谱-质谱检测

将各组检测样品在纳升超高效液相色谱系统（Ultimate RSLCnano 3000，美国Dionex公司）中进行色谱分析，柱温箱温度为50 ℃，流速为500 μL/min，梯度洗脱程序：在65 min内调控流动相B由5%升至60%（V/V）。质谱分析采用四级杆轨道阱质谱（Q Exactive plus，美国Thermo Scientific公司），喷雾电压为2.4 kV，毛细管温度为320 ℃，质谱扫描方式首先采用谱图计数法对不同实验组样本中可能存在的差异肽段进行鉴定及初步筛选，而后采用非数据依赖型数据采集技术（data-independent acquisition, DIA）对初步筛选结果进行定量分析和再次验证。

### 1.4 数据处理分析

首先使用Proteome Discoverer软件将质谱文件转换成Mascot软件的输入格式，而后通过Mascot 2.3软件与人类蛋白质组数据库（Swissprot_Human）进行比对，对各组血清样本中的多肽进行鉴定。在Mascot导出的结果中，对每个肽段鉴定到的谱图数进行统计，取各组中每个肽段谱图数的平均值进行比较，按谱图数变化1.5倍及以上为域值，筛选差异目标肽段。在Mascot鉴定结果的基础上，利用Skyline软件抽提出目标肽段的母离子与子离子峰，排除质量偏差较大的子离子/母离子，计算各离子在每组3个混合样品中的CV，CV越小表示重复性越好，以CV≤30%作为筛选阈值，并比较各离子的质谱峰面积，按峰面积变化1.5倍及以上为域值，验证差异目标肽段。

## 2 结果

### 2.1 质谱检测与质量监控

本次实验对3个实验组中9个混合样本进行了质谱检测，为了监控每个样本提取及质谱运行的平行性，在提取前的样本中加入了等量的重标标准肽GLQAQGYGVR，使用DIA数据在Skyline软件中可以直观的看到标准肽在不同样本中的最终含量差异，将标准肽在各样本中的峰面积进行比较，发现各组间无显著差异，提示样本提取及质谱检测的平行性较好。

### 2.2 多肽鉴定及谱图计数结果

通过与人类蛋白质组数据库进行比对，本次实验在9个混合样本中，共鉴定到545个多肽，分别来自于118个蛋白（表 3）。取各组中肽段谱图数的平均值，作为该肽段在本组样本中的谱图数，并以谱图数变化1.5倍及以上（≥1.5或≤0.67）为域值，共筛选出201个差异多肽，作为下一步DIA定量分析的目标肽段。

**3:** 各组样本中多肽的鉴定 Identification of polypeptides in each group

Group	Peptides	Proteins
Normal	191	40
Benign	438	94
Cancer	362	83
Total	545	118

### 2.3 基于DIA技术的定量分析结果

针对Skyline软件得到的目标肽段母离子与子离子的色谱峰，进行人工确认后导出定量数据进行统计分析。将质量偏差大于20 ppm的离子去除，并计算各离子在每组中的CV，以CV≤30%为阈值，以峰面积变化1.5倍及以上为标准，筛选差异多肽。与Normal组相比，Cancer组共发现28个差异表达多肽；与Normal组相比，Benign组共发现34个差异表达多肽；与Benign组相比，Cancer组共发现20个差异表达多肽（表 4）；在各组比较中，满足CV≤30%的差异肽段共有7个，分别来自于中间α球蛋白抑制因子H4蛋白（ITIH4）、基质γ-羧基谷氨酸蛋白（MGP）、高迁移率族蛋白N2（HMGN2）、胸腺细胞同种异形抗原（TTHY）、胶原蛋白4α亚基（CO4A）、纤维蛋白原α链（FIBA）蛋白（表 4），其中在各组间呈趋势表达变化的有2条肽段，分别是Cancer组中表达下调的ITIH4水解肽段QGAKIPKPEASFSPR和Cancer组表达上调的MGP水解肽段CDDYRLC。

**4:** 各组样本中差异多肽片段的数量 The number of differentially expressed polypeptides in each group

CV	Cancer/Normal	Benign/Normal	Cancer/Benign
≤30%	3	5	1
≤40%	7	9	5
≤50%	18	20	14
Total	25	34	20

## 3 讨论

肺癌是世界范围内常见的一类恶性肿瘤，其发病率、病死率一直居高不下^[9]^肺癌患者的病理分型中85%属于NSCLC，其临床治疗原则为“早发现、早诊断、早治疗”。大多数早期发病的NSCLC患者（TNM分期：Ⅰ期-Ⅱ期），在根治性手术治疗后，都能获得较好的预后。但NSCLC通常具有发病隐匿、进展快速、缺乏特异性肿瘤标志物等特点^[10]^，尤其是针对NSCLC人群的早期筛查，尚缺乏能够用于临床诊断的血清标志物。因此，关于筛选NSCLC早期生物标志物的研究，对于提高其生存率和改善其预后，都将具有十分重要的意义。

多肽组学是一门针对多肽链结构、功能及其相互关系的新兴组学技术，其填补了蛋白组学与代谢组学之间的空隙，正逐渐受到医学界的高度重视^[11, 12]^。越来越多的科研人员尝试通过多肽组学分析，对各类疾病的生物标志物进行筛选和鉴定，其应用领域涵盖肝癌、乳腺癌、卵巢癌、胃癌、膀胱癌等多种恶性肿瘤以及风湿、哮喘和阿尔兹海默症等非肿瘤疾病^[13-17]^。DIA是目前世界上最前沿的非标记型、兼具蛋白质鉴定和相对定量功能的质谱分析技术，其最大优点是无缝的采集所有碎片信息，避免传统数据采集扫描时的数据丢失，且由于DIA可以根据各种离子（母离子与子离子）强度综合评定差异多肽含量，较仅分析母离子的传统数据采集技术更为精确和全面^[15]^。目前，关于NSCLC早期筛查的多肽组学研究还未见文献报道，因此，本实验采用液相色谱结合高分辨质谱技术，利用谱图计数法对不同实验组中可能存在的差异肽段进行鉴定及初步筛选，使用较宽松的筛选条件，舍弃在大部分样本中没有鉴定到或不存在明显差异的肽段，而后通过DIA技术对初筛的差异多肽进行准确的定量分析，从而对初步的鉴定结果进行明确验证，旨在探寻NSCLC早期诊断的血清生物标志物。

本研究发现，在各组比较中，满足峰面积CV≤30%（最严格的筛选模式）的差异肽段共有7个，分别来自于ITIH4、MGP、HMGN2、TTHY、CO4A、FIBA蛋白，而这些蛋白大多被证实是肿瘤组织中重要的调控蛋白。我们实验组会在以后的试验中进一步研究和探讨。其中最具有临床指导意义的是在各组间呈趋势变化的2条肽段，分别是在Normal组、Benign组及Cancer组中表达逐渐降低的肽段QGAKIPKPEASFSPR和表达逐渐升高的肽段CDDYRLC，二者分别来源于ITIH4和MGP蛋白。

ITIH4是一种由肝脏合成的调控细胞外基质分泌和形成的重要血清糖蛋白，属于胰蛋白酶抑制物家族。目前发现，ITIH4异常低表达可能是多种癌细胞恶性转化的重要标志。研究表明，肝癌细胞中ITIH4表达水平显著降低，而过表达调控后可以显著抑制肝癌细胞的迁移，并且发现ITIH4高表达水平的肝癌患者通常具有较好的预后^[16]^，提示ITIH4与肝癌的进展和预后密切相关。本实验的结果也提示ITIH4从正常人到良性疾病再到肺癌中表达逐渐降低，提示其低表达是NSCLC的一种标志，于上述实验结果基本一致。可能的原因是ITIH4在肿瘤组织中的调控作用可能与其裂解肽段有关，其羧基端的多聚脯氨酸区域常出现大量蛋白裂解片段，而这些裂解过程主要参与对细胞外基质的水解破坏，使细胞外基质的结构由于降解而塌陷，从而形成癌细胞恶性侵袭的通道，使其大量通过并浸润到癌旁组织中，最终促成肿瘤细胞的侵袭和转移^[17]^。

目前认为，MGP是一种抑制矿盐沉积的蛋白，可以通过与骨形态蛋白2（BMP2）结合，抑制其诱发的成骨反应和血管钙化^[18]^，而MGP的生物学功能主要依赖于其肽段水解后的非磷酸化修饰^[19]^。近期的研究发现，BMP2与肺癌细胞的侵袭转移密切相关^[20]^，提示MGP也可能是一种肺癌生物标志物。Zandueta等^[21]^研究表明，在人骨肉瘤细胞的肺转移过程中，MGP的表达水平显著升高，且MGP通过激活基质金属蛋白酶活性和TGFβ诱导的Smad2/3磷酸化信号通路，参与调控内皮细胞粘附和上皮细胞-间充质转化，从而促进肿瘤细胞的侵袭转移。与上述研究一样，本实验的研究结果也提示在NSCLC中MGP的表达明显升高，提示可能是一种NSCLC的肺癌标记物。

**5:** 各组样本中峰面积变异系数≤30%的差异多肽 The differentially expressed polypeptides with the CV of peak area less than 30%

Peptide sequence	Protein	Cancer/Normal	Benign/Normal	Cancer/Benign	Cancer/Benign/Normal
QGAKIPKPEASFSPR	ITIH4	Y	Y	Y	Y
CDDYRLC	MGP	Y	Y	Y	Y
RLSAKPAPPKPEPKPK	HMGN2	Y	--	--	--
SYSTTAVVTNPKE	TTHY	--	Y	--	--
NGFKSHALQLNNRQ	CO4A	--	Y	--	--
SYKMADEAGSEADHEGTHST	FIBA	--	Y	--	--
QFTSSTSYNRGDST	FIBA	--	--	Y	--
Y: The difference was statistically significant (*P* < 0.05).					

综上所述，ITIH4与MGP蛋白的水解过程可能对肿瘤疾病的发生发展具有重要的调控作用，其裂解修饰的特异性肽段QGAKIPKPEASFSPR和CDDYRLC可能是潜在NSCLC患者血清中重要的生物标志物，可用于早期肺癌临床筛查，但由于本实验是基于肺癌多肽组学的初步研究，在样本量、病理分型等方面的局限性，实验结果还需进一步的研究验证。
